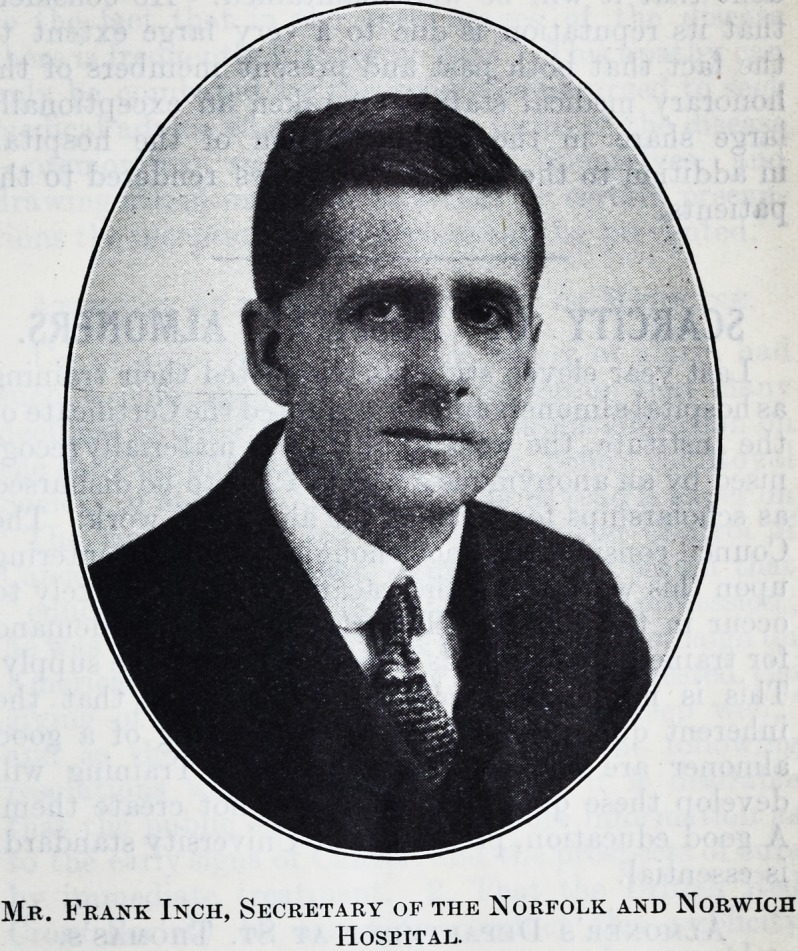# Hospital Men of Mark: Col. A. C. Dawson, C.B.E., and Mr. Frank Inch

**Published:** 1924-10

**Authors:** 


### HOSPITAL MEN OF MARK.

### COLONEL A. C. DAWSON, C.B.E., AND MR. FRANK INCH.

The Norfolk and Norwich Hospital follows the somewhat unusual practice of changing its chairman every year, and during the past forty years very few members of the Board of Management have held the position for more than twelve months. Colonel A. C. Dawson, C.B.E., is one of the exceptions, and it is remarkable that twenty-one years should have elapsed between his two periods of chairmanship. Colonel Dawson first joined the Board of Management in 1898, and in 1903 was elected to the chair.

Col. A. C. Dawson, C.B.E., Chairman of the Norfolk and Norwich Hospital.

During his first term of office the Nurses' Home, containing eighty rooms, generously given by the late Earl of Leicester, was opened, and an indication of the development of the hospital since that date is afforded by the fact that he is now appealing, during his second term, for money to extend the Nurses' Home to double the accommodation provided in 1903.

During the past twenty-one years Colonel Dawson has witnessed the growth of the hospital in all directions. Whereas in 1903 there was a daily average of only 164 patients, with no X-ray, massage, electro-therapeutic or pathological departments, there are now 300 beds, with up-to-date special departments, and an extension scheme is in hand to provide a new out-patient department, children's block of 30 beds, ophthalmic block of 20 beds, maternity wards and 80 additional rooms in the Nurses' Home. To complete this extension scheme a sum of £60,000 is required, and the hospital is fortunate in possessing Colonel Dawson as chairman at this juncture, because his popularity in the county will undoubtedly have a very favourable influence on the success of the appeal for this amount. To Colonel Dawson is due the distinction of instituting the Income Augmentation Committee of the hospital, a body which on many occasions has assisted greatly in keeping things going. In recognition of the many services rendered by him to the Hospital he was elected a vice-president in 1910.

Mr. Frank Inch, the secretary, is a native of Bristol and spent the first seven years of his business life as a clerk to the secretary of the Children's Hospital in that city. Leaving Bristol in 1908 he obtained a post as clerk at the Cardiff Infirmary, and two years

Mr. Frank Inch, Secretary of the Norfolk and Norwich Hospital.

later he was appointed assistant secretary. In 1913 Mr. Inch was appointed secretary of Walsall General Hospital, a position which he held for three years, when he obtained his present position at Norwich. It will be seen that Mr. Inch (who is not yet 40) has had a varied hospital experience extending over a period of twenty-two years. Going to Norwich in March, 1916, he found plenty of scope for his energies, for the Norfolk and Norwich Hospital had undertaken a large task in the admission of sick and wounded soldiers from France. The number of beds provided was increased rapidly, so that eventually there were 274 reserved for soldiers, in addition to the ordinary civilian accommodation. During the war 96 convoys, containing 7,880 men, were admitted from overseas in addition to a large number of patients from among the troops stationed in the district.

Following the war the reorganisation of the hospital was taken in hand, and during the past five years £46,000 has been expended on extensions and alterations. The pathological, X-ray, massage and electro-therapeutic departments have all been reconstructed, a new central heating scheme has been installed, extensions to the laundry carried out, a new home for the domestic servants (with 80 bedrooms) erected, and two electric lifts installed. Valuable additions have been made by the purchase of adjoining properties, and the hospital now has a frontage to the main road of just under 1,000 feet. Concurrently with this large capital expenditure the ordinary income has very materially increased. During the last ten years, indeed, it has risen from £11,427 to £47,284, the Norwich Contributory Scheme being well known as one of the best organised and most productive of the various contributory schemes in existence.

Mr. Inch is proud of the reputation which the Norfolk and Norwich Hospital holds, and feels confident that it will be well maintained. He considers that its reputation is due to a very large extent to the fact that both past and present members of the honorary medical staff have taken an exceptionally large share in the administration of the hospital, in addition to the voluntary services rendered to the patients.

## Figures and Tables

**Figure f1:**
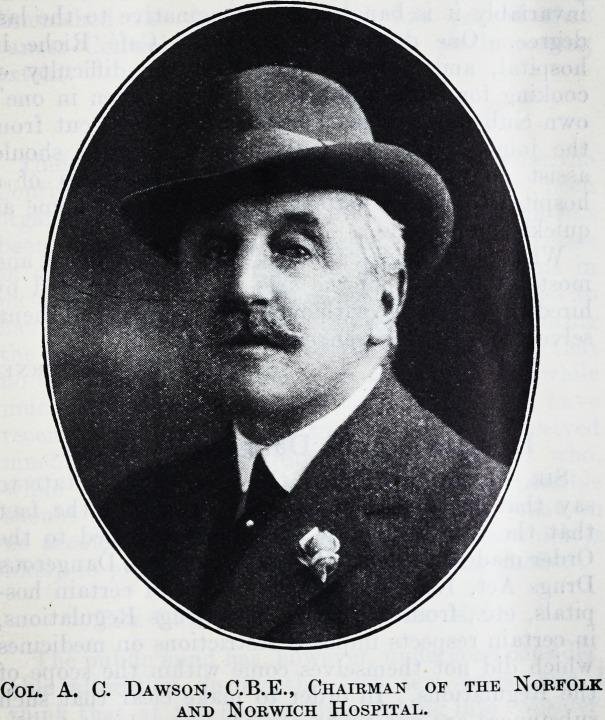


**Figure f2:**